# Microbial aspects and potential markers for differentiation between bacterial and viral meningitis among adult patients

**DOI:** 10.1371/journal.pone.0251518

**Published:** 2021-06-11

**Authors:** Sultan F. Alnomasy, Bader S. Alotaibi, Ahmed H. Mujamammi, Elham A. Hassan, Mohamed E. Ali

**Affiliations:** 1 Department of Medical Laboratories Sciences, College of Applied Medical Sciences in Al- Quwayiyah, Shaqra University, Al- Quwayiyah, Riyadh, Saudi Arabia; 2 Department of Pathology, Clinical Biochemistry Unit, College of Medicine, King Saud University, Riyadh, Saudi Arabia; 3 Department of Gastroenterology and Tropical Medicine, Faculty of Medicine Assiut University, Assiut, Egypt; 4 Department of Microbiology and Immunology, Faculty of Pharmacy, Al-Azhar University, Assiut, Egypt; Public Health England, UNITED KINGDOM

## Abstract

**Objectives:**

Meningitis is a medical emergency with permanent disabilities and high mortality worldwide. We aimed to determine causative microorganisms and potential markers for differentiation between bacterial and viral meningitis.

**Methodology:**

Adult patients with acute meningitis were subjected to lumber puncture. Cerebrospinal fluid (CSF) microorganisms were identified using Real-time PCR. PCT and CRP levels, peripheral and CSF-leucocyte count, CSF-protein and CSF-glucose levels were assessed.

**Results:**

Out of 80 patients, infectious meningitis was confirmed in 75 cases; 38 cases were bacterial meningitis, 34 cases were viral meningitis and three cases were mixed infection. Higher PCT, peripheral and CSF-leukocytosis, higher CSF-protein and lower CSF-glucose levels were more significant in bacterial than viral meningitis patients. *Neisseria meningitides* was the most frequent bacteria and varicella-zoster virus was the most common virus. Using ROC analyses, serum PCT and CSF-parameters can discriminate bacterial from viral meningitis. Combined ROC analyses of PCT and CSF-protein significantly improved the effectiveness in predicting bacterial meningitis (AUC of 0.998, 100%sensitivity and 97.1%specificity) than each parameter alone (AUC of 0.951 for PCT and 0.996 for CSF-protein).

**Conclusion:**

CSF-protein and serum PCT are considered as potential markers for differentiating bacterial from viral meningitis and their combination improved their predictive accuracy to bacterial meningitis.

## Introduction

Infectious meningitis is one of the major lethal infections of the central nervous system causing 422,900 deaths and 2628,000 patients with disabling sequelae globally [1]. A wide range of potentially fatal pathogens can cause infectious meningitis including bacteria, viruses, fungi and parasites that vary in each geographical area and in different age groups [2–4].

Bacterial meningitis is a life-threatening infection with 1.2 million cases each year resulting in 135,000 deaths [5]. Despite the vaccination strategies, antibiotic therapy and good care facilities, the mortality and morbidity rates of bacterial meningitis are still high in both developing and developed countries. On the other hand, viral meningitis usually has a good prognosis and get cured within one or two weeks without any therapy [6]. It is not always possible to differentiate between bacterial and viral meningitis that contributes to excessive empirical use of antibiotics leading to increase their resistance [7]. Furthermore, bacterial meningitis may carry a socioeconomic burden including duration of hospitalization and high financial costs. So, early differentiation between bacterial and viral meningitis, including higher index of suspicion of infection, and the clarification of diagnostic criteria, together with appropriate antibiotic use, have greatly improved the prognosis of these patients, and reduced mortality [5].

Several biomarkers have been proposed to differentiate bacterial from viral meningitis e.g., bacterial antigen testing of cerebrospinal fluid (CSF) and biological markers in the blood including white blood cell [WBC] count and procalcitonin (PCT), or CSF-protein, glucose level, WBC count and CSF-C-reactive protein (CRP) [8–11].

Owing to vaccination strategies, the age distribution of meningitis has now shifted to older age groups [8]. Several studies [9–11] on clinical features and prognostic factors in adults with meningitis have been performed however, to the best of our knowledge, there is no sufficient data regarding the meningitis in our locality.

Therefore, we aimed to identify microbial causative agents of meningitis in adults and assess to clinical and laboratory data to differentiate between bacterial and viral meningitis.

## Materials and methods

This is cross-sectional study was carried out at Faculty of pharmacy, Al-Azhar University-Assiut from March 2017 to December 2018. The study protocol was approved by the local ethics committee of the Faculty of Medicine, Al-Azhar University, Assiut, Egypt and was conducted in according to the ethical guidelines of the Declaration of Helsinki. A written informed consent was obtained from all participants or from relatives for patients with disturbed consciousness.

This study consecutively included adult patients with acute meningitis admitted to Assiut Fever Hospital, Assiut, Egypt between March 2017 and December 2018. The diagnosis of meningitis was suspected when the patients had the following clinical features of meningeal irritation: headache, fever, neck stiffness, photophobia, altered conscious level and focal neurological signs (The diagnosis of meningitis was suspected when the patients had the following clinical features of meningeal irritation: headache, fever, neck stiffness, photophobia, altered mental status (assessed using the Glasgow come scale; where, altered mental status was defined as a score on the Glasgow coma scale ≤14) and focal neurological signs including Cranial nerve palsies, aphasia, monoparesis or hemiparesis) [12, 13]. We excluded patients who received antibiotic therapy or immunomodulating agents and who were less than 18 years old.

At the study entry, patients were subjected to thorough medical history and physical examination. Lumbar punctures (LP) were performed and CSF specimens were submitted for laboratory analysis including leucocyte count, protein and glucose level and for PCR analysis. Blood sample was taken for complete blood count, CRP and PCT.

## Methods

With universal safety precautions and standard laboratory protocols, 3–4 ml of CSF was collected into sterile screw-cap tubes each containing one ml and the blood sample was collected into a plain tube for serum separation.

### DNA extraction

Total DNA was extracted from CSF using Gentra Puregene Tissue Kit (QIAGEN, USA). Absorbance spectrophotometry was used for check DNA purity, quality and quantity (Nanodrop-1000; Nanodrop Technologies, Wilmington, DE, USA). Finally, DNAs were stored at –20°C for further processing.

### Real-time PCR

Real time PCR was performed using SYBR Green (Bioline, USA) and ABI 7500 Sequence Detection System (Applied Biosystems). Each microorganism RT-PCR detection reaction was carried out in simplex PCR out to record its exact melt peaks. Cycling conditions and primer sequences were set as described before in previous studies [14–18], PCR amplification reactions were performed with 12.5μl of SYBR Green Supermix, 1μl of the primers (10 pmol) shown in Table 1, 3μl of template DNA and deionized water was used to make up the total volume to 20μl.

**Table 1:** Primers used in this study.

Microorganism	Primer	Amplicon melting temperature (°C)
Varicella zoster virus (VZV)	F: 5’-TGAGGGGATAGCTAAAATCG-3’ R: 5’-TATAAAAGTTTTTTCACACTC-3’	79
Cytomegalovirus (CMV)	F: 5’- ATAGGAGGCGCCACGTATTC-3’ R: 5’- TACCCCTATCGCGTGTGTTC-3’	84
Enterovirus (EV)	F: 5’- GGCCCCTGAATGCGGCTAAT-3’ R: 5’- ATTGTCACTGGATGGCCAAT -3’	85
Herps simplex 1 (HSV-1)	F: 5’- CCATACCGACCACACCGACGA-3’ R: 5’- CATACCGGAACGCACCACAC -3’	92
Herpes simplex 2 (HSV-2)	F: 5’-TACGCTCTCGTAAATGCTTC -3’ R: 5’- GCCCACCTCTACCCACAA T -3’	92
Human endogenous retrovirus 3 (HERV-3)	F: 5’-CATGGGAAGCAAGGGAACTAATG -3’ R: 5’-CCCAGCGAGCAATACAGAATTT -3’	83
Epstein Barr virus (EBV)	F: 5’- GCCGGTGTGTTCGTATATGG -3’ R: 5’- CAAAACCTCAGCAAATATATGAG -3’ F: 5’- GGAGATACTGTTAGCCCTG -3’ R: 5’- GTGTGTTATAAATCTGTTCCAAG -3’	62
*N*.*meningitides*	F: 5’-TGTGTTCCGCTATACGCCATT -3’ R: 5’- GCCATATTCACACGATATACC -3’	87
*S*.*pneumonia*	F: 5’-GAATTCCCTGTCTTTTCAAAGTC-3’ R: 5’-ATTTCTGTAACAGCTACCAACGA-3’	85
*H*.*influenzae*	F: 5’- GCACTTCTGGAATTAACGC -3’ F: 5’- AGGGCTATTGCAGCAAACTT -3’	60
*P*.*aeruginosa*	F: 5’-AGTTGTCGCGGCGCTACTAC-3’ R: 5’-GCTCACCTGGATCTGGTCCA-3’	94
*S*.*aureus*	F: 5’-TCGGTACACGATATTCTTCAC-3’ R: 5’- ACTCTCGTATGACCAGCTTC-3’	80

*N.meningitides: Neisseria meningitides. S.pneumonia: Streptococcus pneumonia. H.influenzae: Haemophilus influenzae. P.aeruginosa: Pseudomonas.aeruginosa. S.aureus: Staphylococcus.aureus*.

### Serum procalcitonin level determination by ELISA

Serum procalcitonin level determination by ELISA according to the manufacturer’s instructions (RayBiotech, Peachtree Corners, GA).

### C-reactive protein level determination by ELISA

C-reactive protein level determination by ELISA according to the manufacturer’s instructions (RayBiotech, Peachtree Corners, GA).

### Statistical analysis

All statistical analyses were carried out using SPSS for Windows version 16 (IBM Corp., Armonk, NY, USA) and the MedCalc program. The Shapiro-Wilk Test of Normality was used to test the normality of data. Quantitative variables were expressed as mean ± standard deviation or median and the range (minimum—maximum) and were compared using Student’s t or Mann-Whitney U-tests for normally or abnormally distributed data, respectively. Qualitative variables were expressed as percentage and compared using chi-squared (χ 2) or Fisher’s exact probability test. Spearman’s rank correlation coefficient (r) was used to find correlations. The receiver operating characteristic (ROC) curves were plotted to measure and compare the performance of different parameters to discriminate bacterial from viral meningitis and to select the best cut-off point at which sensitivity, specificity, positive (PPV) and negative (NPV) predictive value, positive and negative likelihood ratio (+LR, −LR) were calculated. All tests were two-tailed and statistical significance was assessed at < 0.05.

## Results

### Characteristics of the studied patients

**Fig 1:**
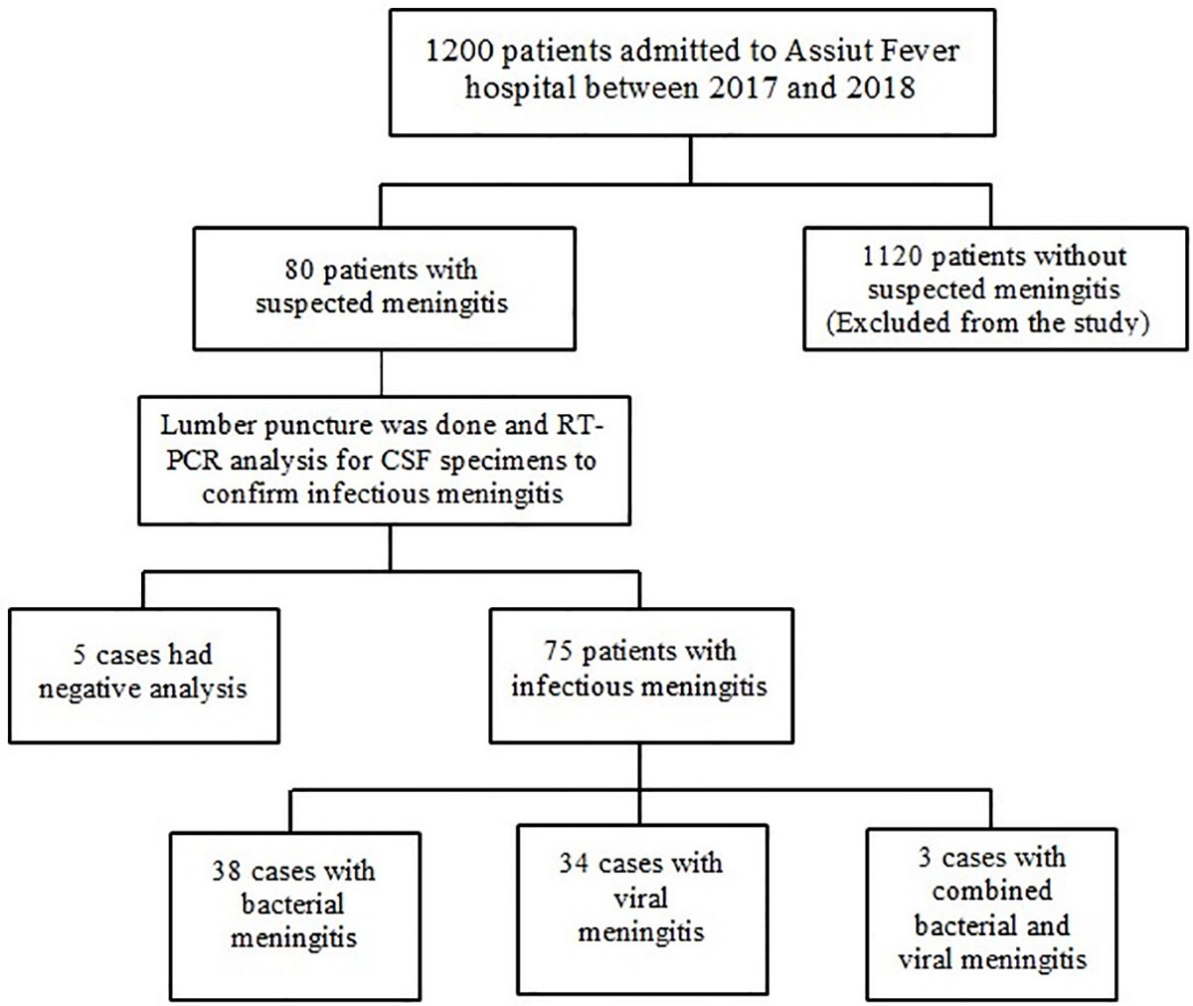
The patient flow chart in the study.

A total of 80 patients with suspected meningitis (38 males and 42 females with mean age of 37.3 ± 10.3 years) admitted to Assiut Fever Hospital, Assiut, Egypt between March 2017 and December 2018 were enrolled in the study. Fever and severe headache were the most common symptoms and presented in 84% of patients. Of those 80 patients, infectious meningitis was confirmed from CSF aspirates using RT-PCR in 75 (93.8%) cases (33 males and 42 females with mean age of 37.4 ± 10.6 years) where 38 cases were bacterial meningitis, 34 cases were viral meningitis and three cases were combined bacterial and viral meningitis (1 male and 2 females with mean age of 38± 12.3 years). The remaining five patients (3 females and 2 males with mean age of 35 ± 10.6 years) had negative analysis as shown in flow chart in Fig 1.

In patients with bacterial meningitis, smoking and comorbidities (underlying medical conditions that can affect health status and immune response of patients and hence they facilitate acquiring infections including diabetes mellitus (DM), obesity and chronic debilitating diseases like renal, hepatic and others) including diabetes mellitus and obesity were significantly higher compared to patients with viral meningitis. Apart of severe headache, clinical manifestations were more significant in bacterial than viral meningitis patients. In addition, patients with bacterial meningitis had significantly higher levels of WBCs, PCT, CSF-leucocyte count and CSF-protein than those with viral meningitis. On the other hand, CSF-glucose levels were significantly lower in bacterial than viral meningitis patients. The sociodemographic characteristics and clinical data of these patients were summarized in Table 2.

**Table 2:** Sociodemographic and clinical data of patients with infectious meningitis.

	Total patients with infectious meningitis* (n = 75)	Patients with bacterial meningitis (n = 38)	Patients with viral meningitis (n = 34)	P
Age (years)	37.4 ± 10.6 (21–61)	38.6 ±10.7 (21–57)	38.5±10.4 (21–61)	0.444
Sex (Male/Female) (n, %)	33/42	22/16 (57.9/42.1%)	11/23 (32.4/67.6%)	0.06
Smoking (n, %)	26 (34.7%)	20 (52.6%)	6 (17.6%)	0.002
Comorbidities (n, %)	19 (25.3%)	14 (36.8%)	5 (14.7%)	0.033
High grade fever (n, %)	63 (84%)	36 (94.7%)	27 (79.4%)	0.05
Severe headache (n, %)	63 (84%)	34 (89.5%)	29 (85.3%)	0.592
Neck stiffness (n, %)	28 (37.3%)	22 (57.9%)	6 (17.6%)	< 0.001
Altered mental status (n, %)	20 (26.7%)	17 (44.7%)	3 (8.8%)	0.001
Photophobia (n, %)	23 (30.7%)	19 (50%)	4 (11.8%)	0.001
Focal neurological signs (n, %)	32 (42.7%)	26 (68.4%)	6 (17.6%)	< 0.001
CSF-Leucocyte count (cell/mm^3^)	5 ± 2	7 ± 3	3 ± 1	< 0.001
CSF-protein (g/dl)	1.8 ± 1	2.8 ± 1.3	0.6 ± 0.3	< 0.001
CSF-glucose (mmol/l)	10 (1–54)	4 (1–13)	20.3 (3–54)	< 0.001
WBCs (cell/mm^3^)	4800 (698–12300)	6750 (1600–12300)	2245 (698–9800)	< 0.001
Serum CRP (mg/l)	23.3 ± 9.9	25 ± 10.8	22.3 ± 8.6	0.238
Serum PCT (ng/ml)	1.4 (0.2–5)	2.4 (1–5)	0.8 (0.2–3.4)	< 0.001

Values are presented as mean ± standard deviation or n (%) unless otherwise indicated. P-value <0.05 means significant.

* 75 patients included 38 cases of bacterial meningitis, 34 cases of viral meningitis and the cases were combined viral and bacterial meningitis.

### Causative agents of meningitis in this study

By real time PCR analysis of CSF samples of those patients, 41 CSF samples were positive for bacterial DNA, 37 samples were positive for viral DNA where, three samples were positive for mixed infections. For bacterial meningitis, *N*.*meningitides* was the most frequent bacteria followed by *S*.*pneumonia* and *H*.*influenzae*. For viral meningitis, VZV was the most common followed by EV and HSV-1 three samples were positive for mixed infections (bacterial and viral), one of them containing *S*.*aureus* and EV, other containing *S*.*aureus* and VZV, last one containing *P*. *aeruginosa* and VZV. The details of microbial infections among patients with meningitis were shown in Table 3.

**Table 3:** Causative agents of meningitis in this study.

	Bacterial meningitis (n = 38)	Viral meningitis (n = 34)	Mixed (Bacterial +Viral) (n = 3)
***S*. *aureus***	0	-	2
***P*. *aeruginosa***	0	-	1
***H*. *influenzae***	8	-	
***S*. *pneumonia***	11	-	
***N*. *meningitides***	18	-	
**VZV**	-	15	2
**HERV-3**	-	0	
**HSV-1**	-	8	
**HSV-2**	-	3	
**EV**	-	7	1
**CMV**	-	1	

*N*.*meningitides*: *Neisseria meningitides*. *S*.*pneumonia*: *Streptococcus pneumonia*. *H*.*influenzae*: *Haemophilus influenzae*. *P*.*aeruginosa*: *Pseudomonas*.*aeruginosa*. *S*.*aureus*: *Staphylococcus*.*aureus*. Varicella zoster virus (VZV), Cytomegalovirus (CMV), Enterovirus (EV), Herps simplex 1 (HSV-1), Herpes simplex 2 (HSV-2), Human endogenous retrovirus 3 (HERV-3), Epstein Barr virus (EBV).

### Correlation between CSF parameters and blood parameters

In viral meningitis, CSF protein significantly correlated with WBCs (r = 0.353, P = 0.041) and CSF glucose levels correlated with CRP (r = 0.550, P = 0.001). Regarding bacterial meningitis, no correlations were found between different parameters.

#### Predictive accuracy and determination of the best cut-off value of CRP, PCT and combine (CRP, PCT) cells for discriminating bacterial from viral meningitis

**Fig 2:**
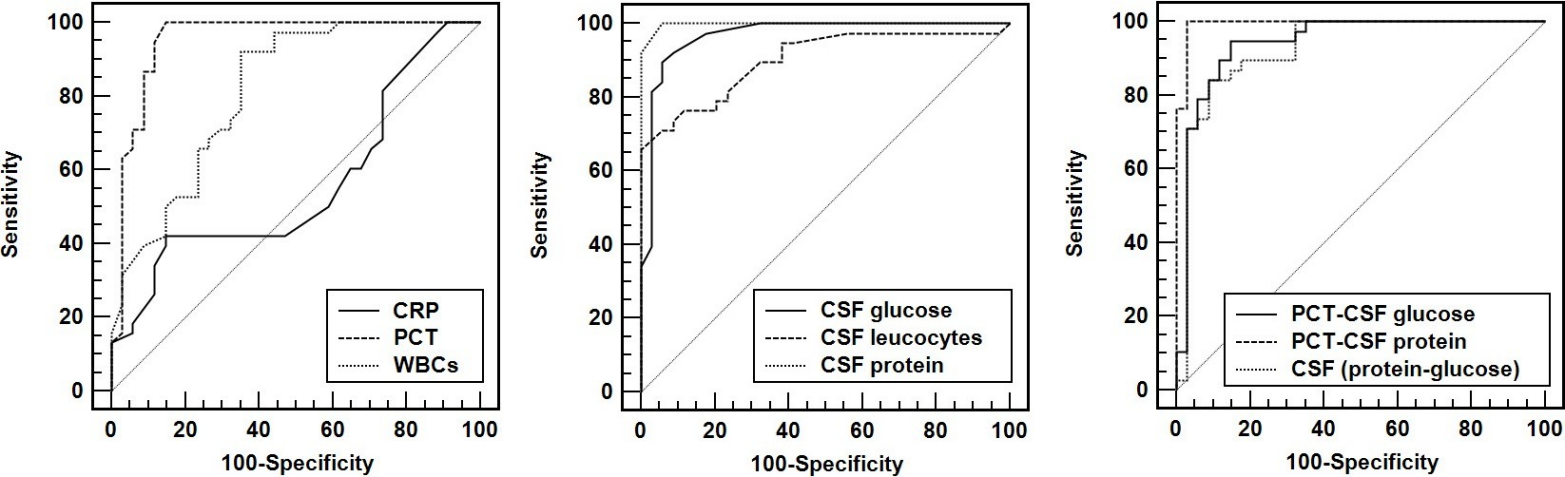
Receiver operating characteristic curve of prognostic parameters for the discriminating bacterial from viral meningitis.

ROC analyses revealed that PCT and WBCs may be helpful to discriminate bacterial from viral meningitis, with an area under the ROC curve (AUC) of 0.951 (95% confidence interval (CI) = 0.873–0.988) for PCT and 0.815 (95%CI = 0.706–0.897) for WBCs. At a cut-off value of >0.98 ng/m) for PCT, the sensitivity was 100% and the specificity was 85.3%. On the other hand, at the cut-off value of >3480 cell/mm^3^ for WBCs, the sensitivity was 92.1% and the specificity was 64.7% (Table 4 and Fig 2A). In addition, ROC curve analysis revealed that CSF levels of leucocytic count, protein and glucose were potential biomarkers for discriminating patients with bacterial meningitis from viral meningitis where CSF protein had the largest AUC of 0.996 (95% CI = 0.945–1) with a sensitivity of 100% and a specificity of 94.1% at a cut-off value of >6 x 10^9^/l (Table 4 and Fig 2B). Further, combined ROC analyses of PCT and CSF protein significantly improved the effectiveness in differentiating bacterial from viral meningitis with the largest AUC [0.998 (95%CI = 0.937–0.999), P < 0.001), a sensitivity of 100% and specificity of 97.1% (Table 4 and Fig 2C).

**Table 4:** Diagnostic accuracy of prognostic parameters to discriminate bacterial from viral meningitis with the best predictive cut-offs.

Variables	AUC 95% CI	SEN (%)	SPE (%)	PPV (%)	NPV (%)	+LR	-LR
CSF leucocyte count (> 622.5 cell/mm^3^)	0.901 (0.808–0.959)	65.8	100	100	79.8	-	0.3
CSF protein (> 1 g/dl)	0.996 (0.945–1)	100	94.1	92.6	100	17	0
CSF glucose (< 9.8 mmol/l)	0.967 (0.985–0.994)	84.2	94.1	91.3	89	15.2	0.1
CRP (>29 (mg/l))	0.560 (0.438–0.677)	42.1	85.3	67.9	66.6	2.9	0.7
PCT (> 0.98 ng/ml)	0.951 (0.873–0.988)	100	85.3	83.4	100	6.8	0
WBCS (> 3480 cell/mm^3^)	0.815 (0.706–0.897)	92.1	64.7	65.9	91.7	2.7	01
PCT-CSF protein	0.998 (0.937–0.999)	100	97.1	96.2	100	34	0
PCT-CSF glucose	0.941 (0.859–0.98)	94.7	85.3	82.6	95.6	6.4	0.1
CSF glucose and protein	0.926 (0.840–0.974)	84.2	91.2	87.6	88.6	9.5	0.2

AUC: area under the curve; SEN: sensitivity; SPE: specificity; PPV: positive predictive value; NPV: negative predictive value; +LR: positive likelihood ratio; -LR: negative likelihood ratio.

## Discussion

Unlike viral meningitis, bacterial meningitis can have devastating consequences and can be lethal if not promptly diagnosed or treated at an early stage. Early assessment of meningitis is a highly challenge for a physicians’ practice to improve the management and decrease the mortality. This study aimed to highlight the epidemiological and microbial aspects of infectious meningitis and to demonstrate potential markers for differentiation between bacterial and viral meningitis.

Our analysis identified infectious meningitis in 75 of 80 patients with clinically suspected meningitis where the majority of cases had bacterial meningitis (50.7%) followed by viral meningitis (45.3%). This higher frequency of bacterial meningitis was within the range of the previous estimates (47%-68%). Fouad et al., (2014) identified bacterial meningitis in 73.3% of the studied patients [6]. Unlike our finding, several studies revealed the higher prevalence of viral than bacterial meningitis; Dawod et al., (2019) reported that viral meningitis represented 87.1% compared to 12.8% bacterial meningitis of the studied population [19]. Águeda et al., (2013) declared that viral infection was responsible for the majority of meningitis (52.9%) [20].

Similar to previous studies [21, 22], we found that smoking and comorbidities e.g., DM and obesity were significantly associated with bacterial meningitis. Tobacco smoke exposure increases the risk of meningococcal disease however obesity and diabetics raise the risk of pneumococcal meningitis [21–23]. These risk factors can indirectly lead to acquisition of meningitis by their negative effect on immune function and host defense mechanisms and susceptibility to infection [21, 24, 25].

In this study, neck stiffness, disturbed level of consciousness and focal neurologic signs were more significant in bacterial (57.9%, 44.7% and 68.4%, respectively) than viral meningitis patients (17.6%, 8.8% and 17.6%, respectively). These manifestations are related to the severity of meningitis and the time interval before hospitalization [26]. Our results were in agreement with those of Dawod et al., [19] and Agueda et al., [20] which showed that *N*. *meningitides* was the most frequent bacteria followed by *S*. *pneumonia* and *H*. *influenza* causing bacterial meningitis. However, other studies reported *S*. *pneumoniae* to be the leading causative agent followed by *N*. *meningitidis* and *H*. *influenzae* by culture and by real-time PCR [27–30]. On the other hand, coagulase-negative staphylococci species were the prevalent causative agent for acute bacterial meningitis that primarily affected adults in Qatar hospitals [31]. In the last century, *N*. *meningitidis* was identified as the main etiological agent for bacterial meningitis [32, 33]. Later, N. *meningitidis* was defined as the second or third leading cause after *S*. *pneumoniae* [27, 28, 34].

For viral meningitis, VZV was the most common followed by EV and HSV-1. Franzen-Röhl, et al., (2007) reported that HSV-2 was found by real-time PCR in CSF from 80% of patients compared to 72% found by nested PCR followed by VZV (4.4%) [35]. However, Akkaya et al., (2017) demonstrated that Enterovirus was the most frequently identified agent followed by Adenovirus and *S*. *pneumoniae* [36]. The discrepancy in the causative agents among different studies may be due to different age groups and different geographical area.

In this study, serum PCT levels were significantly higher in bacterial that viral menigitis. In addition, PCT had higher performance in discriminating bacterial form viral meningitis compared to WBCs and CRP (AUC = 0.951) and at a cut-off value of 0.98 ng/ml, with sensitivity and NPV of 100%. Our findings were consistent with earlier studies [37, 38]. Abdelkader et al., (2014) reported higher values of serum PCT at admission and at 3 days post-treatment in bacterial than viral meningitis [39]. Makoo et al., (2010) stated that serum PCT had a cut off of 0.5 ng/ml with sensitivity and NPV of 100% suggesting that serum PCT test can be a useful marker in differentiation between bacterial and viral meningitis [40]. Moreover, Alkholi et al., (2011) revealed that serum PCT with a cut off value >2 ng/ml showed sensitivity [41], specificity, positive predictive value, and negative predictive value of 100%, 66%, 68%, and 100% respectively for the diagnosis of bacterial meningitis.

We found that peripheral WBC count was significantly higher with good diagnostic performance (AUC = 0.815) in bacterial than viral meningitis patients with high sensitivity and NPV at a cut off value of > 3480 cell/mm^3^. Ray et al., (2007) reported that plasma inflammatory markers e.g., peripheral WBC count can be very useful in distinguishing bacterial from nonbacterial meningitis [42].

Unlike previous studies [43–45], we found that serum CRP had poor diagnostic performance to discriminate bacterial from viral meningitis. This may be due to its low levels in bacterial meningitis in our study which could be attributed to measuring serum CRP within the first hours of suspected meningitis. Our findings were compatible with earlier studies reporting that CRP may show a delayed increase during the course of bacterial infection, leading to false-negative tests in the early stages of the disease [46–48]. CRP can also be increased in viral infections which limits its ability to distinguish between bacterial and viral meningitis [49, 50].

In consistence with Fouad et al., (2014), we found that significant CSF-lecucytosis, higher protein levels and lower glocuse levels in bacterial meningitis patients compared to viral meningitis group. However, some studies revealed that the CSF profile alone could not reliably discriminate between bacterial and non-bacterial meningitis [51, 52]. In this study, validation of significant CSF parameters showed that CSF-protein level had the largest AUC (0.996) with the 100% sensitivity, 94.1% specificity, 92.6% PPV and 100% NPV at a cutoff vlue of >1g/dl in discriminating bacterial from viral meningitis. Several studies reported the similar finding with variation in the mean values between bacterial and aseptic meningitis patients [6, 53].

Based on the data in this study, we found that a coupling of serum PCT and CSF-protein had a superior diagnostic performance (AUC = 0.998, P < 0.001) to predict bacterial meningitis than each parameter alone achieving rapid, simple and easily applicable tool that could guide a clinical decision in an emergency setting till culture results appearance. This model has yet to be validated prospectively. As this study was a single-center study with small sized sample, further multicenter studies on larger scales are needed to confirm these findings and to help guide early diagnosis and treatment strategies in those high risk patients.

In conclusion, serum PCT, CSF lecucytosis, CSF-protein and CSF-glucose together with signs of meningeal irritation and neurological signs can discriminate bacterial from viral meningitis where, CSF-protein has the highest diagnostic accuracy. Coupling CSF-protein and serum PCT significantly improves their predictive accuracy to expect bacterial meningitis that may aid in rapid diagnosis and quality of care improvement till culture results appearance and hence in the reduction of mortality.

## Supporting information

S1 File (XLSX)
